# Transfer of Perceptual Learning From Local Stereopsis to Global Stereopsis in Adults With Amblyopia: A Preliminary Study

**DOI:** 10.3389/fnins.2021.719120

**Published:** 2021-09-24

**Authors:** Adrien Chopin, Michael A. Silver, Yasha Sheynin, Jian Ding, Dennis Michael Levi

**Affiliations:** ^1^School of Optometry, University of California, Berkeley, Berkeley, CA, United States; ^2^Département d’Etudes Cognitives, Ecole Normale Supérieure, Paris, France; ^3^Sorbonne Université, INSERM, CNRS, Institut de la Vision, Paris, France; ^4^Helen Wills Neuroscience Institute, University of California, Berkeley, Berkeley, CA, United States; ^5^Vision Science Graduate Group, University of California, Berkeley, Berkeley, CA, United States; ^6^McGill Vision Research Unit, McGill University, Montréal, QC, Canada

**Keywords:** stereopsis, amblyopia, perceptual learning, stereoblindness, depth perception, local stereopsis, global stereopsis, amblyopia treatment in adults

## Abstract

It has long been debated whether the analysis of global and local stereoscopic depth is performed by a single system or by separate systems. Global stereopsis requires the visual system to solve a complex binocular matching problem to obtain a coherent percept of depth. In contrast, local stereopsis requires only a simple matching of similar image features. In this preliminary study, we recruited five adults with amblyopia who lacked global stereopsis and trained them on a computerized local stereopsis depth task for an average of 12 h. Three out of five (60%) participants recovered fine global stereoscopic vision through training. Those who recovered global stereopsis reached a learning plateau more quickly on the local stereopsis task, and they tended to start the training with better initial local stereopsis performance, to improve more on local stereopsis with training, and to have less severe amblyopia. The transfer of learning from local stereopsis to global stereopsis is compatible with an interacting two-stage model.

## Introduction

Stereopsis, the rich percept of depth that we get through binocular disparity (i.e., the difference between the two eyes’ views of the world), provides critical information about the distances between objects in the scene. A longstanding question is whether there are (at least) two separate systems that process binocular disparity, one local and the other global (Julesz, 1978; Ross, 1983; Ptito et al., 1991; Gantz and Bedell, 2010).

Local stereopsis is based on matching similar image features in the two eyes (Julesz, 1978; Vancleef et al., 2017). Global stereopsis (Julesz, 1960), on the other hand, is more complex, as it requires the visual system to solve a complex binocular correspondence problem (for example, correctly matching pairs of binocular dots in a large array of similar dots) to obtain a coherent percept of depth. In global stereograms, typically random dot stereograms (RDSs), shapes that are defined by depth cannot be seen before the correspondence problem is solved, and after that, illusory contours appear. In local stereograms, typically contour stereograms, solving the corresponding problem is not necessary to see the shapes because the contours that define the shapes are visible.

Evidence for two separate systems for stereopsis comes from studies of the effects of cortical lesions. In some patients, global stereopsis is impaired while local stereopsis survives (Hamsher, 1978; Ptito et al., 1991). However, this result (Hamsher, 1978; Ptito et al., 1991) was not obtained in another study (Ross, 1983). Ptito et al. (1991) reported impaired global but intact local stereopsis in patients with lesions in the anterior temporal cortex. Hamsher (1978) found global stereopsis defects for right hemisphere lesion patients with intact local stereopsis. However, Hamsher tested local stereopsis with the Titmus circles test, which allows for correct responses using monocular cues (Simons and Reinecke, 1974; Chopin et al., 2019b).

Conclusive evidence in favor of the two-systems hypothesis would require a double dissociation. However, to date, only impaired global stereopsis with intact local stereopsis has been reported. In addition, it is possible that global stereopsis was worse than local stereopsis before any lesion, as global stereopsis requires more complex computations. Global and local stereopsis performance are not correlated across participants (Gantz and Bedell, 2010), even when tested under the same conditions with a similar stimulus, suggesting two separate mechanisms.

An alternative hypothesis is that there is a single mechanism comprised of two mutually interacting stages. At one stage, the binocular correspondence problem is solved, and this solution can be used at a second stage, where disparities are extracted for depth perception. Processing of local stereograms involves primarily the disparity extraction stage whereas processing of global stereograms (e.g., RDSs) relies heavily on both stages. Therefore, any deficit at the stage of solving the correspondence problem will result in lower global stereoacuity without necessarily impairing local stereopsis. Variation in the ability to solve the correspondence problem will also lead to low correlations between local and global stereoacuities across individuals. This is in line with evidence from a study in which adaptation to global stereograms transfers to local stereopsis (Rose and Price, 1995). One interpretation of this finding is that the disparity extraction stage is adapted by the global stereograms. In addition, Gantz and Bedell (2010) reported bidirectional transfer of perceptual learning between local (low density) and global (high density) RDSs and concluded that there is a single mechanism for stereopsis. However, they noted: “…because perceptual learning has been shown to be specific for orientation (Karni and Sagi, 1993), spatial frequency (Sowden et al., 2002), and other stimulus properties (Fahle, 2005), it remains unknown if training on contour stereograms would transfer to RDSs with substantially different spatial characteristics, or vice versa.” Indeed, perceptual learning with stereograms is strongly specific to the stimulus properties such as the orientation of its elements (Ramachandran and Braddick, 1973).

Here we show that improvements in perceptual learning of local stereopsis based on training with contour stereograms can transfer to global stereopsis as measured with RDSs. The transfer of learning from local to global stereopsis supports an interacting two-stage model.

## Materials and Methods

We conducted an exploratory study of the effects of training on a local stereopsis task on fine global stereorecovery in five participants with amblyopia.

### Participants

We recruited adult observers with amblyopia from patients at the UC Berkeley Optometry Eye Clinic who met the following inclusion criteria: at least a two-line interocular acuity difference with best-corrected vision after excluding any eye disease, with a clear amblyogenic cause for their amblyopia (strabismic if any tropia was present, anisometropic if any refraction difference >1 spherical-equivalent diopter was present, or mixed if both criteria were present).

Exclusion criteria included strabismus angle >30Δ, monocular visual acuity worse than 20/200, age >65 years, binocular amblyopia, nystagmus, intermittent exotropia, diplopia, a history of traumatic brain injury or severe neurological or psychiatric disorder, current psychoactive medications, language barrier, any MRI contraindication (we intended to scan their brain), and already being recruited in another training experiment. Only 30 participants from an initial pool of 955 (Supplementary Figure 1) were eligible and interested in participating. After testing participants with the Diplopia-Suppression test (described below), four were excluded because we could not obtain fusion in the best conditions. After testing participants with the Eyetracked RDS (described below), 18 were excluded, either because they already had fine global stereoscopic acuity (<900″) or because they dropped out before beginning the training. Data from five participants who completed the training are reported here (mean age: 29.6, 80% female, 2 with strabismic amblyopia, 1 with anisometropic amblyopia, and 2 with mixed amblyopia). Their clinical details are provided in Table 1. Two additional participants completed the training but were later shown to have global stereopsis before training, following a more thorough analysis, and were excluded. One additional participant dropped out. The study followed the tenets of the Declaration of Helsinki and was approved by the Committee for the Protection of Human Subjects at the University of California, Berkeley (protocol #2013-08-5542) and by the CERES at Paris-Descartes (ethics committee–N°IRB: 20151100001072).

**TABLE 1:** Participant’s clinical information.

Code	Age/Sex	Amblyopia type/eye	Refraction at 3 m distance	Snellen acuity Pre-training	Snellen acuity Post-training	Tropia	Comment	Training days/hours/trials
012	23/F	Mixed/OS	OD: Plano−0.50 × 165 OS: +2.75−1.00 × 155	OD: 20/20−2 OS: 20/100+1	OD: 20/20+1 OS: 20/100+1	Dist.: 8ΔLXT Near: 4ΔLXT		18/15.7/11,683
013	26/M	Strabismic/OD	OD: +1.75−1.75 × 180 OS: +1.25−2.00 × 180	OD: 20/160−1 OS: 20/20−1	OD: 20/160−1 OS: 20/20−1	8ΔRHyperT		10/9.1/6,832
014	25/F	Anisometropic/OS	OD: −0.25−0.75 × 180 OS: +4.50−5.25 × 180	OD: 20/20+2 OS: 20/63+2	OD: 20/16+1 OS: 20/63+1	Ortho		15/14.1/8,820
015	30/F	Strabismic/OS	OD: +0.25−0.50 × 45 OS: −0.50−1.00 × 143	OD: 20/25−1 OS: 20/80	OD: 20/20+2 OS: 20/100+1	Dist.: Ortho Near: 2ΔLET	Microstrabismic	13/10.8/9,100
016	44/F	Mixed/OS	OD: Plano OS: +2.25−2.00 × 70	OD: 20/12.5−1 OS: 20/50−1	OD: 20/16−2 OS: 20/50−2	Ortho	Microstrabismic (from 4ΔBO test)	11/8.4/4,900

### Procedure

We conducted an interventional preliminary study. After an initial screening session, we tested participants on the Randot stereotest. Then, if they could pass the Diplopia-Suppression Test (which determines whether observers are capable of fusion without suppression), we measured their global stereoacuity with the Eyetracked RDS test, for near and far disparities, at two different stimulus durations. If they had stereoacuity worse than 900″ for near and far disparities, and for all durations, we invited the participants to undergo stereorecovery training for an average of 11.6 h in the task (10–18 sessions of 1–1.5 h). We trained their local stereopsis with contour stereograms that contained no monocular cues to depth. After the training, we retested stereoacuity with the Randot stereotest and the Eyetracked RDS test.

#### Randot Clinical Stereotest

We tested participants on the Randot stereotest (modified version, also called version 2, produced by Stereo Optical Co., Inc.), once in the standard orientation and once with an orientation that was rotated 90°. When rotated, the test contains the same disparities, but they are vertical and do not provide useful stereoscopic depth information. If a participant had the same performance or better with the rotated test compared to the standard orientation, we concluded that there was no stereopsis and that the participant was using binocular non-stereoscopic cues to perform the test (Chopin et al., 2019b).

The Shapes part (global stereopsis) and Circles part (local stereopsis) were recorded separately for the Randot test, and we followed the manufacturer’s instructions. However, for the Randot Shapes portion, these instructions are unclear on the number of shapes necessary to score one level of disparity. We scored a given level if three out of four shapes (or absence of shape) were correctly recognized. We tested each level independently and recorded the best score.

#### Diplopia-Suppression Test

All participants had to pass the Diplopia-Suppression Test before we tested their stereoacuity with the Eyetracked RDS stereotest. The Diplopia-Suppression Test determines whether participants can obtain fusion while avoiding diplopia and interocular suppression in the stereoscope. It is valid for the current stereoscope configuration only. For each participant, the stereoscope mirror positions were first adjusted to equalize accommodation and vergence distances, as these can be altered when viewing stimuli through a stereoscope. For that purpose, participants horizontally aligned a line that was presented monocularly on the screen and viewed through the mirrors with a line, drawn on a wood stick, that was presented above the screen and viewed directly.

After this calibration, we presented a black-and-white frame to both eyes, with a monocular circle and a binocular fixation dot in the middle of the frame. Half of the monocular circle was presented to one eye, and the other half was presented to the other eye. Observers adjusted the relative vertical locations of the stimulus presented to each eye so that the binocular frame appeared to be fused and the two monocular half circles appeared to be vertically aligned. The interocular contrast, and if necessary, the horizontal location, could also be varied (with one eye’s contrast fixed at 96%) if the binocular frame was not fused or if one of the two half-circles was suppressed by the other.

Next, a binocular central circle replaced the monocular half-circles, and six smaller circles were presented, three on each side of the central circle. In some of the small circles (min: 0 and max: 2), one binocular dot was presented. Participants were asked to report the number of dots perceived in each of 10 trials, and these reports were used to compute the proportion of trials with diplopia (i.e., when the number of perceived dots was twice the number of actual dots).

Finally, participants were asked to detect whether one dot was present or not in each of the six circles. The dot could be monocular in the left eye (15 trials) or monocular in the right eye (15 trials). We used the responses to calculate a detection sensitivity d’ for each eye and considered it a measure of each eye’s interocular suppression. If less than 20% of the trials were diplopic and all d’ values were above one, we considered the participants to have passed the Diplopia-Suppression test, and we saved the interocular stimulus locations and contrasts for that participant for use in the other tasks. For each trial of the test, each keypress produced a sound. If participants could not achieve fusion at a distance of 75 cm, the test was repeated at a distance of 150 cm. If participants still could not achieve fusion, we excluded them from the study.

#### Eyetracked Random Dot Stereogram Stereotest

The Eyetracked RDS test is a computerized test of global stereopsis that is based on dynamic RDSs. In this test, the relative disparity of a background square around a central target square is varied to measure the participant’s stereoacuity. The squares are defined solely by the binocular disparities of the dots and have no visible contours.

**FIGURE 1:**
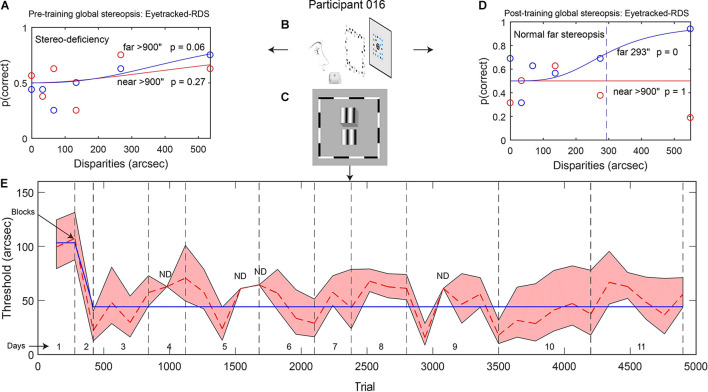
**(A,D)** Example data from participant 016: psychometric functions showing global stereopsis performance before training (**A:** stereo-deficiency) and after training (**D:** normal stereopsis for far disparities) on the Eyetracked RDS task with a 200-ms stimulus presentation. The circles depict the proportion of correct trials for each relative disparity between target and background (red: near disparities, blue: far disparities). Red and blue continuous lines represent model fits (based on Eq. 1), and the dashed line indicates the threshold disparity value for far disparities after local stereopsis training. **(B,C)** Stimuli used for measuring global stereopsis (**B**, Eyetracked RDS) and for training local stereopsis (**C**, contour stereograms). **(E)** Local stereopsis thresholds across training trials. The red dashed line shows changes in thresholds that were calculated for each block of trials. Gray vertical dashed lines separate training days. The blue continuous line indicates thresholds from the best model (derived from Eq. 4). The red shaded area depicts the SEM (ND: SEM was not defined).

The Diplopia-Suppression Test yielded values of relative interocular locations and the interocular contrast difference that were optimal for fusion for each participant. The Eyetracked RDS stimuli were centered in each eye at the Diplopia-Suppression-Test’s locations and using its contrast values for the fusion frames and fixation dot in each eye. The task consisted in using keypresses to report whether the background square was in front of or behind the target square on each trial (Figure 1B). We enforced fixation on the target (which was in the same depth plane as the fixation point) using eye tracking, thereby removing the possibility that participants would employ the delta vergence strategy (i.e., using changes in vergence to infer the change in depth; Backus and Matza-Brown, 2003). Each trial started with a fixation dot and circle, and fusion frames. The participant pressed a key to extinguish the fixation dot and circle and to initiate stimulus presentation, which was followed by presentation of a blank screen until the participant responded. Each keypress produced a tone. The method of constant stimuli was used to vary the disparities of the background square (relative to the target square) while the absolute disparity of the target square remained at zero. We measured thresholds twice, once with a stimulus presentation duration of 200 ms and once with a duration of 2000 ms.

Before data collection, participants were given several practice trials. All of the practice trials had relevant auditory feedback (high-frequency beep for correct responses, low-frequency beep otherwise), and practice trials were repeated if the participant did not reach 61% correct. For the first practice, we presented two repeats of each large disparity (137.5″, 275″, 412.5″, 550″, and 1100″, near and far) with a 2000-ms stimulus duration. For the second practice, we presented two repeats of each large disparity with a 1000-ms stimulus duration. For the third practice, we measured two repeats of each test disparity (0″, 34.5″, 69″, 137.5″, 275″, and 550″, near and far) with a 1000-ms stimulus duration. For the fourth practice, we measured two repeats of each test disparity with a 200-ms stimulus duration.

After practice was complete, we presented 16 repeats of each test disparity with a 200-ms stimulus duration and presented an irrelevant auditory stimulus (the same frequency on every trial) after each response. From the responses to these stimuli, we calculated the 200-ms stereoacuity for near and far disparities separately.

In a second session, participants again completed the Diplopia-Suppression Test, followed by practice trials in which we trained participants to fixate exclusively inside the fixation circle during 2000-ms trials. We used their eye position to provide real time visual feedback with a dot whose color was green when close to the fixation dot and red when close to the fixation circle limit. If participants fixated outside of the circle, the trial was interrupted, a loud sound was presented, and the trial was repeated. In a second practice session, we instructed the participants to perform both the fixation task with visual feedback and the Eyetracked RDS task (two repeats of each test disparities) for 2000-ms trials.

Finally, we administered 16 repeats of each test disparity for 2000-ms trials, with no visual feedback and irrelevant auditory feedback. Participants were instructed to fixate only inside the fixation circle. Gaze was tracked on every other trial, and if participants moved their eyes outside the circle, the trial was interrupted, a loud sound was presented, and the trial was repeated. From the responses to these stimuli, we calculated the 2000-ms stereoacuity for near and far disparities separately. Final stereoacuity was defined as the better of the 200- and 2000-ms stereoacuities, separately for the near and far disparities. Participants could take breaks every 10 min.

#### Stereo Training

Participants were trained with a contour stereogram task (Figure 1C). The training consisted of 10–18 sessions with durations between 1 and 1.5 h, resulting in an average of 11.6 h of training per participant on the main task (ranging from 8.4 to 15.7 h and from 4,900 to 11,680 trials; see Table 1). These values only reflect training time and do not include stereoscope preparation time or instructions. Each training session started with the Diplopia-Suppression Test. In the rare cases in which fusion was not possible, we invited the participant to come back for another visit.

After the Diplopia-Suppression Test, which started the training, participants trained on the main task. In this task, participants reported which of two vertical gratings was in front of the other. Each trial started with the fixation stimulus. We instructed participants to align the upper and lower monocular fixation lines as much as possible (achieved by verging in the fixation depth plane) on each trial and to press a key when alignment was achieved. The keypress caused the removal of the fixation stimulus and initiated continuous presentation of the two gratings until a response was made. Auditory feedback then indicated whether the response was correct. Eye movements were not restricted, and participants were encouraged to use all possible means to be successful in the task. In each session, participants completed 3–5 blocks of 20 repeats of seven different disparities (10 trials with the upper grating in front and 10 with the lower in front). Participants could take breaks every 10 min.

### Stimuli

#### General Parameters

The gray background luminance was 15 cd/m^2^, white was 30 cd/m^2^, and black was around 0.5 cd/m^2^. Anti-aliasing allowed subpixel precision for stimulus display.

#### Diplopia-Suppression Test

Fusion frames were squares with length of 9° of visual angle, with a 3° diameter central circle, inside of which was a black fixation dot. The fixation dot had a diameter of 0.2°. Each side of the fusion frames was composed of four line segments, alternating black and white. The six black small circles were 0.6° in diameter, while the six black dots inside the circles were 0.25° in diameter. The contrast of the frames and fixation dot seen by the dominant eye was varied during testing, with the weaker eye’s contrast fixed at 96%.

#### Eyetracked Random Dot Stereogram Stereotest

A black circle (2.5° diameter) surrounded a black fixation dot (0.25° diameter) and was centered within a white fusion frame (13.6°). The RDS contained a target square (1.6°) in the middle of a larger background square (3.2°), defined only by the binocular disparities of the random dots. Half of the dots of the RDSs were white, and the other half were black (0.25° diameter, 20%-density). However, the non-black dots within the target square were shaded blue (Figure 1B). Only the background-square random dots had a disparity which varied from trial to trial (0″, 34.5″, 69″, 137.5″, 275″, and 550″, near and far); all other displayed elements were presented at 0″-fixation disparity. Every 100 ms, a new set of random dots were presented with the same disparity configuration. This dynamic RDS was necessary to prevent the alternating fixation strategy (monitoring the direction of the horizontal shift of a visual item while alternately closing one eye, to deduce depth ordering).

#### Stereo Training

Fusion frames were 13.6° squares with a 2.2° diameter central circle, inside of which was a fixation dot. Each side of the fusion frames was composed of four alternating black and white line segments (0.3° wide). The fixation stimulus consisted of a binocular dot, two binocular horizontal lines, and two monocular vertical lines. The fixation dot was 0.25° in diameter. The lines were 0.5° in length, forming a disjointed cross around the fixation dot. All the stimuli were presented at location values obtained for each participant from the Diplopia-Suppression Test as corresponding to the center for each eye. The contrast values of the fusion frames and fixation dot and circle in each eye were also obtained from the Diplopia-Suppression Test.

The contour stereograms were comprised of two vertical gratings, located one above the other, separated by some depth (Figure 1C). Each grating was 3° × 3° in size and contained sinusoidal luminance modulation (spatial frequency: 1 cpd) embedded in a Gaussian envelope along the horizontal axis (full width at half maximum: 1.5°). The phase of each modulation was randomized and was different for each grating, thereby preventing the use of an alternating fixation strategy. A gap of 0.33° separated the two vertically aligned gratings. The phase disparity between the gratings spanned the following seven values: 10″, 63″, 117″, 170″, 223″, 277″ and 330″, with half of the disparity in the upper grating and the other half in the lower grating. The upper grating was in front for half of the trials.

### Material

Matlab and PsychToolbox were used to generate all stimuli with a Macintosh Power PC computer. Stimuli were presented on a NEC SuperBright Diamondtron MultiSync FP2141 monitor (1600 × 1200 pixels; 39 × 29.5 cm; refresh rate 60 Hz) at a viewing distance of 75 cm through a custom-made four-mirror stereoscope. Eyetracking was achieved with an Eyelink II eyetracker running on a separate computer and using ViewPoint software. We customized the calibration code so that it could run with half a screen, given that only half the screen was available to each eye through the stereoscope. We designed the Eyetracked RDS test to be free of non-stereoscopic cues, and we used a threshold of 900″ (15′) to denote total stereoblindness.

### Analysis Procedure

#### Eyetracked Random Dot Stereogram Stereotest

Eyetracked RDS data were analyzed separately for the 2000 and 200-ms trials and for the near and far disparities. The final Eyetracked RDS stereoacuity was the best demonstrated stereoacuity in any of the conditions that was significantly different from the stereo-deficient model.

For each observer, we calculated the 75% threshold value using a method that is independent of lapse rate or stereo deficiency that is exclusive to near or far disparities. We expressed the probability of a correct response as a function of the disparity between the target and background, separately for near and far disparities (zero disparity trials were included in both near and far disparity conditions). We modeled these data with a logistic psychometric function of log-disparity (Serrano-Pedraza et al., 2016), containing two parameters that vary independently: threshold and slope.


(1) $$\Psi \left(x,\theta \right)=g+\frac{1-l-g}{1+exp\left(-b\left[a+x-\theta \right]\right)}$$

(2) $$b=\frac{2}{\sigma }\ln\left(\frac{1-l-g-d}{d}\right)$$

(3) $$a=\frac{1}{b}\ln\left(\frac{1-l-p}{p-g}\right)$$

Ψ is the percentage of correct responses.

*x* is the base-10 logarithm of the presented disparity.

*p* is the performance level defined to be the threshold (75%).

θ is the detection threshold in log-disparity: the value of *x* at which equals *p*.

*l* is the lapse rate: the probability of error at maximum disparity (set at 1.75%).

*g* is the guess rate: the probability of chance performance (50% for two-alternative choices).

σ is the spread parameter, or slope, quantifying how performance increases with disparity.

*d* represents the top/bottom of the psychometric function (we use 1%).

We derived the parameter values of the best model fit by employing a chi-square minimization procedure. The threshold parameter θ was bounded between 8.6″ and 3000″, and the slope parameter σ was bounded between 0.2 and 2.4. The parameters that produced the smallest chi-square after a hundred repetitions of the procedure with random initial parameter settings were selected for the stereo-typical model.

We also tested a competing stereo-deficient model, identical to the stereo-typical model except that θ was fixed at 3000″. Again, the parameters that produced the smallest chi-square after a hundred repetitions with random initial parameter settings were selected. We carried out a chi-square test (with 1° of freedom) to test whether the best chi-square for the stereo-typical model was significantly different than the best chi-square for the stereo-deficient model. If not, we did not exclude the stereo-deficient model and assigned a threshold of 900″ and stereo-deficiency for that condition. We did the same for thresholds at 900″ or above.

#### Stereo Training

We calculated the percentage of correct responses as a function of the disparity between the two gratings for each block (20 trials for each of the seven disparities). We extracted the threshold for each block following the same procedure as above for the Eyetracked RDS.

We then modeled changes in contour stereogram thresholds across trials with the probit-derived function in Eq. 4 applied to the logarithm of the thresholds, following normalization of the data:


(4) $$F=T_{1}+\left(1-T_{end}-T_{1}\right)*0.5*\left(1+\mathrm{erf}\left(\frac{t-T_{i}}{\sqrt{2}\alpha }\right)\right)$$

With $T_{t}=\frac{\theta t}{\text{log}\left(2000\right)}$ (5)

Here, θ_*t*_ is the log threshold in arcsec at trial *t*, *T*_1_ is the log normalized threshold at trial 1, *T*_*end*_ is the log normalized threshold at the last trial of the training, *T*_*i*_ is the log normalized threshold at the inflection point, and α is the learning rate. All of the above parameters were free and estimated through chi-square minimization. We modeled the data with both F (increasing function) or 1-F (decreasing function) and then kept the model with the best chi-square value. Our procedure allows us to account for a potential initial period during which thresholds were stable before beginning to increase or decrease.

After fitting the thresholds that were obtained for each block of the training, we could extrapolate the threshold values θ_1_ and θ_*end*_ for the first and last trial of the training, respectively. An example of the resulting model fit is provided in Figure 1E.

For all participants, we found rapid learning transitions from initial performance levels, followed by a plateau. We manually identified the trial of the first block at plateau performance (local stereopsis plateau trial).

#### Statistics

All Bayes factors for two-sample comparisons were calculated using the Bayes Factor Toolbox^1^, with the function bf.ttest2 when investigating differences between paired groups. For all parametric tests, normality of distributions was assessed using the Shapiro–Wilk test (all *p* > 0.06, except for the distribution of differences between pre- and post-training local stereopsis, *W* = 0.70, *p* = 0.0098, for which we therefore used a non-parametric test). When not otherwise specified, tests were two-tailed with an alpha level of 5%, and *t*-tests were conducted on paired samples. Hedge’s g formula is the Cohen’s d formula with an unbiased variance estimator. All correlations were calculated using the Pearson formula.

## Results

We quantified local stereopsis performance during the training task, using a probit-derived model that allowed extrapolation of performance at the first and last training trial (see methods). Overall, local stereopsis improved during the training (Wilcoxon signed-rank test: *W* = 15, *p* = 0.031).

**FIGURE 2:**
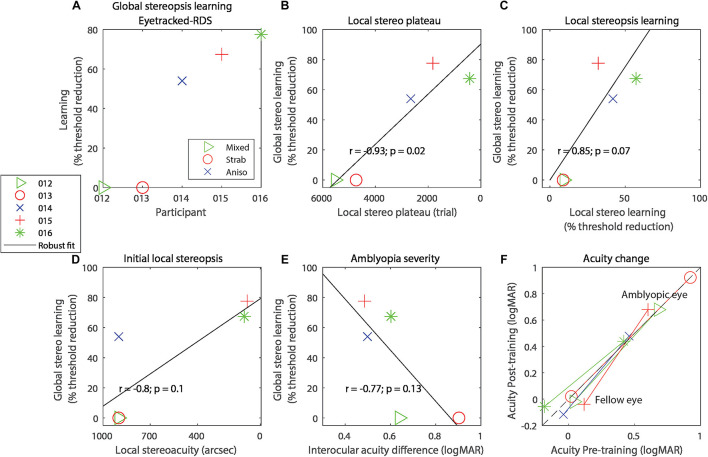
**(A)** Global stereo learning, expressed as percent threshold reduction on the Eyetracked RDS test for each of the participants. On all panels, symbols code for the participant and colors code for the type of amblyopia. **(B)** Correlation between global stereo learning and local stereo plateau trial number. **(C)** Correlation between global stereo learning and local stereo learning. **(D)** Correlation between global stereo learning and performance on the local stereopsis task extrapolated to trial 1. **(E)** Correlation between global stereo learning and amblyopia severity (defined as the interocular acuity difference). **(F)** Visual acuity change with training, expressed in logMAR. Lines connect each eye’s acuity values for the same participant. Data points below the black diagonal dashed line indicate improvement in visual acuity with training. In panels **(B–E)**, the solid diagonal black line represents a robust linear regression following an iteratively reweighted least-squares technique with a bisquare weighting function.

We measured participants’ global stereoacuity with the Eyetracked RDS stereotest before and after training (Figures 1A,D for participant 016). All of the participants were stereo-deficient before training (thresholds > 900″) and were assigned a stereo threshold value of 900″ (Table 2). Learning was expressed as percentage of threshold reduction, or 100^∗^(θ_*Pre*_–θ_*Post*_)/θ_*Pre*_. After training, three out of five participants (60%) achieved fine global stereovision (one-tailed pre–post *t*-test *t*(4) = 2.39, *p* = 0.038, Hedge’s g = 1.07, BF10 = 2.01; Figure 2A), indicating transfer from trained local stereopsis to untrained global stereopsis for these participants.

**TABLE 2:** Stereoacuities pre and post-training.

	Pre-training			Post-training			
Participant	*Randot Shapes/Circles*	Eyetracked-RDS (global stereo)–Near/Far	Contour stereograms (local stereo)*	*Randot Shapes/Circles*	Eyetracked-RDS (global stereo)-Near/Far	Contour stereograms (local stereo)*	Comments
012	Nil/200″	>900″/>900″	>900″	Nil/Nil+	>900″/>900″	816″	No depth feeling
013	Nil/Nil	>900″/>900″	>900″	Nil/Nil	>900″/>900″	821″	No depth feeling
014	Nil/25″	>900″/>900″	>900″	Nil+/Nil+	414″/>900″	523″	No depth feeling pre-training, depth popped out post-training for Eyetracked-RDS but not in everyday life
015	500”/Nil	>900″/>900″	84″	500″/50″	202″/480″	57″	No depth feeling pre-training, depth popped out post-training for Eyetracked-RDS but not in everyday life
016	Nil/Nil	>900″/>900″	103″	Nil/Nil+	>900″/293″	44″	No depth feeling pre-training, popped out during training, transferred post-training to far depth in everyday life when fusion is voluntarily achieved

*+ This participant obtained an equal or better non-nil level with the test rotated 90°, demonstrating their use of binocular non-stereoscopic cues.*

* Thresholds for the first and last trials are calculated from the probit-derived model.

*Nil: null performance–for analyses of Eyetracked-RDS and contour-stereogram results, the value 900″ was used where we indicated >900″.*

Of these three participants, one recovered global stereopsis for both near and far disparities, one for near disparities only, and the other for far disparities only. Among these three participants, the worst measurable post-training global stereoacuity was 414″ (which is well within Panum’s fusion area in central vision; Palmer, 1961), and the best stereoacuity was 202″. Global stereorecovery was not confined to participants with a specific type of amblyopia: one had anisometropic amblyopia, one had strabismic amblyopia, and the third had mixed amblyopia.

In exploratory analyses, we related global stereorecovery to other experimental variables. For each participant, we determined the trial at which a plateau occurred in the local stereopsis learning task. Participants that reached the plateau sooner showed a larger improvement in global stereopsis (Figure 2B, *r* = −0.94, *p* = 0.016, *R*^2^ = 0.89). Three other variables were marginally associated with global stereopsis improvements. Larger global stereo improvements were marginally associated with greater local stereopsis learning on the training task (Figure 2C, *r* = 0.85, *p* = 0.067, *R*^2^ = 0.73), expressed as a percentage of threshold reduction at the last trial relative to the threshold estimated at trial 1, extrapolated with a probit-derived model (see section “Materials and Methods”).

Larger global stereo improvements were also marginally associated with better performance at the first trial of local stereopsis training (Figure 2D, correlation: *r* = −0.80, *p* = 0.10, *R*^2^ = 0.64) and with lower amblyopia severity, defined as the absolute interocular acuity difference (Figure 2E, *r* = −0.77, *p* = 0.13, *R*^2^ = 0.59). Indeed, the two participants who did not recover any global stereopsis had more severe amblyopia than the three participants who recovered, although this difference was only marginally significant (independent-samples one-tailed *t*-test *t*(3) = 2.22, *p* = 0.057, Hedge’s g = 2.02, BF10 = 1.37).

We also explored possible effects of local stereopsis training on visual acuity and found no significant effects (Figure 2F), neither for the fellow eye (*t*(4) = 0.70, *p* = 0.52, Hedge’s g = 0.31, BF10 = 0.55) nor the amblyopic eye (*t*(4) = 1.66, *p* = 0.17, Hedge’s g = 0.74, BF10 = 0.50).

## Discussion

We trained five adults with amblyopia who lacked global stereopsis on a computerized local stereopsis depth task for an average of 12 h. Three out of five participants (60%) recovered fine global stereoscopic vision.

Is it possible that our participants actually had fine global stereopsis but we could not measure it before training? We consider fine disparities to be those within Panum’s area (Chopin et al., 2019a) and coarse disparities as those outside of it, with a limit of 900″. We were very careful to ensure that all participants had deficient global stereopsis, with thresholds above 900″. Specifically, we tested for fine global stereopsis both for near and far disparities, providing both polarities, and under optimal conditions (Tam and Stelmach, 1998; Westheimer, 2013; Chopin et al., 2019a). Specifically, we employed large adjacent stimuli with colored targets, large high contrast dots, long exposures, and measurable fusion with visible frame locks and absence of suppression through contrast and position adjustments. Absence of suppression and diplopia were verified with the Diplopia-Suppression test. We also eliminated non-stereoscopic cues from the measurement of global stereopsis: monocular cues through the use of dynamic RDSs and binocular non-stereoscopic cues with a front-behind protocol (2AFC sign-depth task). Delta vergence was mitigated through brief presentations (too short for vergence movements to occur) or eyetracker-enforced fixation (in the long exposure condition).

Participants underwent a long practice session before testing (112–224 trials). Before training, participant 015 successfully passed the 500″ level on Randot Shapes, which tests for fine global stereopsis. However, good performance on this test can be achieved by using binocular non-stereoscopic cues. Indeed, participant 015 was able to use such cues to reach a score of 250″ with the test rotated 90° (conducted at mid-training). This is why we prefer to use the psychophysical measure for global stereopsis (Eyetracked RDS). Based on this test, we conclude that participant 015 did not have fine global stereopsis.

After training, three out of five participants (60%) acquired fine global stereopsis. Is it possible that the two non-recovering participants simply needed more training to obtain fine global stereopsis? Previous studies have shown that asymptotic learning for depth discrimination of RDSs occurs after approximately 4000–5000 trials (Fendick and Westheimer, 1983; Gantz et al., 2007). We administered more trials than this to the participants who did not recover (for a total of 6,823 and 11,683 trials). However, for visual acuity, participants with more severe amblyopia needed more monocular training to reach asymptotic performances (Li et al., 2008; Levi and Li, 2009). If we apply that rationale to local stereopsis training, we cannot exclude the possibility that the participants in our study who did not achieve fine global stereopsis could have eventually attained this with more training.

The fact that both local and global stereopsis improved after training only local stereopsis is evidence for a transfer of learning from local to global stereopsis. Additional support for transfer is the marginal correlation between the amount of learning for local and global stereopsis across participants.

What can explain the relation between faster plateaus for learning of the local stereopsis task and larger improvements in global stereo? It is possible that the plateau variable captures smaller effects of other variables with which it is strongly correlated. Indeed, faster plateaus were strongly associated with larger local stereo improvements (*r* = −0.94, *p* = 0.016, *R*^2^ = 0.89) and better initial local stereopsis performance (*r* = 0.83, *p* = 0.082, *R*^2^ = 0.69).

There was a trend for both better initial local stereopsis performance and less severe amblyopia to be associated with greater global stereopsis learning. These results are in line with a meta-analysis of amblyopia interventions, in which participants with more severe amblyopia showed less improvement in stereo sensitivity than those with milder amblyopia, and better initial stereopsis predicted stereopsis improvements after treatment (Tsirlin et al., 2015). Our findings are also consistent with a recent study (Portela-Camino et al., 2020) that found that stereo improvements were predicted by initial global stereoacuity, although their study employed global stereopsis training on a sample with stereo-deficiency and treated amblyopia. Given the small sample size that we used in our preliminary study, we cannot definitively conclude that any of these factors had an effect on improvement in untrained global stereopsis.

Only one participant recovered both near and far global stereopsis, with the two others recovering either near or far stereopsis. We enforced fixation with an eyetracker only in the global stereopsis test. In the local stereopsis training task, participants were free to move their eyes and to fixate in any depth plane. Therefore, it is possible that during the local stereopsis task, one participant favored fixation on the far depth plane while another favored fixation on the near plane, thereby creating an imbalance in their near and far stereopsis performances.

Each session of the training includes a Diplopia-Suppression Test that helps calibrating the stereoscope and enforces fusion. Theoretically, passing the Diplopia-Suppression Test could generate changes in suppression that could participate in local and global stereopsis learnings. In practice, it is unlikely to contribute substantially to perceptual learning of stereopsis because it is only a few minutes long. In addition, Vedamurthy et al. (2015) found no significant correlation between changes in suppression and stereopsis improvement during perceptual learning.

Gantz and Bedell (2010) found that learning of local stereopsis transfers to global stereopsis. Our results are compatible with theirs, but in contrast to their study, we used contour stereograms that are typically used to test local stereopsis, rather than low-density random-dot stereograms. Combining our results with those reported in the literature, we conclude that there is likely a single mechanism for stereopsis with two interacting stages. At one stage, a solution for the binocular correspondence problem is selected and at the other stage, binocular disparities are extracted based on this particular solution. The result is propagated back to the first stage to facilitate finding a correspondence solution that favors small overall disparities (Read, 2002). In this way, the two stages are interacting with each other. Gantz and Bedell (2010) concluded from their results that a single mechanism is at play for both local and global stereopsis, and they excluded a two-stage model. This is because they only considered a sequential (feedforward) two-stage model, while we consider an interacting two-stage model.

An alternative but closely related hypothesis is that the visual system has multiple spatial-frequency-selective first-order pathways for luminance signal (carriers) and second-order pathways for contrast signals (envelopes). In this framework, each pathway has two parallel stages for solving the correspondence problem and extracting disparity. The two stages of all pathways work simultaneously with mutual interactions. When a stereogram is presented to the two eyes, an ambiguous disparity profile is first extracted without solving the correspondence problem, resulting in many binocular mismatches. The correspondence problem is more likely first solved at the lower frequencies (Ding and Levi, 2021), resulting in extraction of a clearer disparity profile with fewer mismatches. This clearer disparity profile would further assist in solving the correspondence problem at the smaller scales, resulting in a vivid 3D profile extracted with less mismatches. This is consistent with a recent model for stereopsis (Ding and Levi, 2021) which contains multiple spatial-frequency-selective first- and second-order pathways, each with two processes in parallel for binocular fusion (solving the correspondence problem) and depth perception (extracting stimulus disparity). A stereo-deficient individual could have deficits in first- and/or second-order pathways. Deficits in the second-order pathway may lead to difficulty in solving the correspondence problem, in extracting disparity, or in both, thus leading to failure to detect depth in RDSs that lack first-order local features. On the other hand, a luminance sinewave grating stereogram with sharp contours contains both first- (the grating and contours) and second-order (the envelope) information, so training can improve stereo performance in both pathways, providing a reasonable explanation of why the training effect can transfer from local to global stereograms.

Delta vergence is a component of stereopsis that is based on using vergence eye movements to sequentially estimate absolute disparities (Backus and Matza-Brown, 2003) that are then employed to calculate relative disparity (Chopin et al., 2016). While the contribution of delta vergence to stereopsis is weak, it is not known whether it has a role in perceptual learning of stereovision. In our study, we are unable to address this question, as we only measured global stereopsis without delta vergence, not isolated delta vergence performance before training. During local stereo training, delta vergence could have been used by the participants, because we presented the stimulus continuously until they made a response.

It is possible to obtain local and global stereo improvements through perceptual learning in amblyopia (Astle et al., 2011; Xi et al., 2014; Levi et al., 2015; Vedamurthy et al., 2016). While global stereopsis can be improved with global stereogram training (Astle et al., 2011), it was not previously known whether local stereopsis training could yield the same result. Other studies investigating stereo improvements from local stereopsis training did not measure global stereopsis both before and after training (Ding and Levi, 2011; Xi et al., 2014; Vedamurthy et al., 2016). It is possible that deficits in processing stimuli presented to the amblyopic eye prevent observers with stereo-deficiency from learning how to solve the binocular correspondence problem. Ding and Levi (2011) found that local stereopsis training with monocular cues allowed stereoblind strabismic non-amblyopic participants to recover local stereopsis when monocular cues were removed. In Ding and Levi’s study, it is not clear how global stereopsis was affected, because participants were only tested for global stereopsis after training. Here, we show that an almost identical training procedure, but without monocular cues, allows amblyopic (including non-strabismic) participants with partially spared local stereopsis to recover fine global stereopsis.

Does stereorecovery lead to a decrease in the severity of amblyopia? The rationale behind that hypothesis is that stereopsis relies on visual information from an eye that is usually suppressed in amblyopia. Therefore, stereorecovery could lead to better binocular cooperation and a decrease in binocular acuity difference (Levi and Li, 2009). In our study, neither the training, nor the improvements in stereopsis, resulted in increases in visual acuity. However, we note that our sample size was too small to be conclusive with respect to this question. Nevertheless, our findings are consistent with past results (Ding and Levi, 2011; Vedamurthy et al., 2016) and with work showing that monocular spatial acuity training resulted in visual acuity improvement in two people with amblyopia but that additional global stereopsis training only improved global stereoacuity and not visual acuity (Astle et al., 2011).

Li et al. (2016) showed that learning transfers more efficiently from high to low spatial frequencies than from low to high frequencies. This could limit learning in strabismic observers who have difficulty processing high spatial frequencies in a stereoacuity task (Ding and Levi, 2011). Here, we show transfer of stereoacuity in people with amblyopia from low spatial frequencies to a broadband stimulus.

In conclusion, we document recovery of fine global stereopsis in people with amblyopia after training local stereopsis using contour stereograms. The transfer of learning from local to global stereopsis is compatible with an interacting two-stage model.

## Data Availability Statement

Data and codes described in this study are available on the OSF repository at https://osf.io/nsy4v with DOI 10.17605/OSF.IO/NSY4V.

## Ethics Statement

The study was approved by the Committee for the Protection of Human Subjects at the University of California, Berkeley (protocol #2013-08-5542) and by the CERES at Paris-Descartes (Ethics Committee–N°IRB: 20151100001072). The patients/participants provided their written informed consent to participate in this study.

## Author Contributions

All authors contributed to conception and design of the study, manuscript revision, read, and approved the submitted version. AC and YS collected the data. AC and DL analyzed the data and wrote the manuscript.

## Conflict of Interest

The authors declare that the research was conducted in the absence of any commercial or financial relationships that could be construed as a potential conflict of interest.

## Publisher’s Note

All claims expressed in this article are solely those of the authors and do not necessarily represent those of their affiliated organizations, or those of the publisher, the editors and the reviewers. Any product that may be evaluated in this article, or claim that may be made by its manufacturer, is not guaranteed or endorsed by the publisher.

## Footnotes

1
https://klabhub.github.io/bayesFactor/

## Supplementary Material

Supplementary Figure 1 Diagram of the recruitment procedure and results.
