# Supplementary figures and images for: Transfer of Perceptual Learning From Local Stereopsis to Global Stereopsis in Adults With Amblyopia: A Preliminary Study
Source: Front Neurosci. 2021 Sep 24;15:719120. doi: 10.3389/fnins.2021.719120 (PMC8498040; doi:10.3389/fnins.2021.719120)

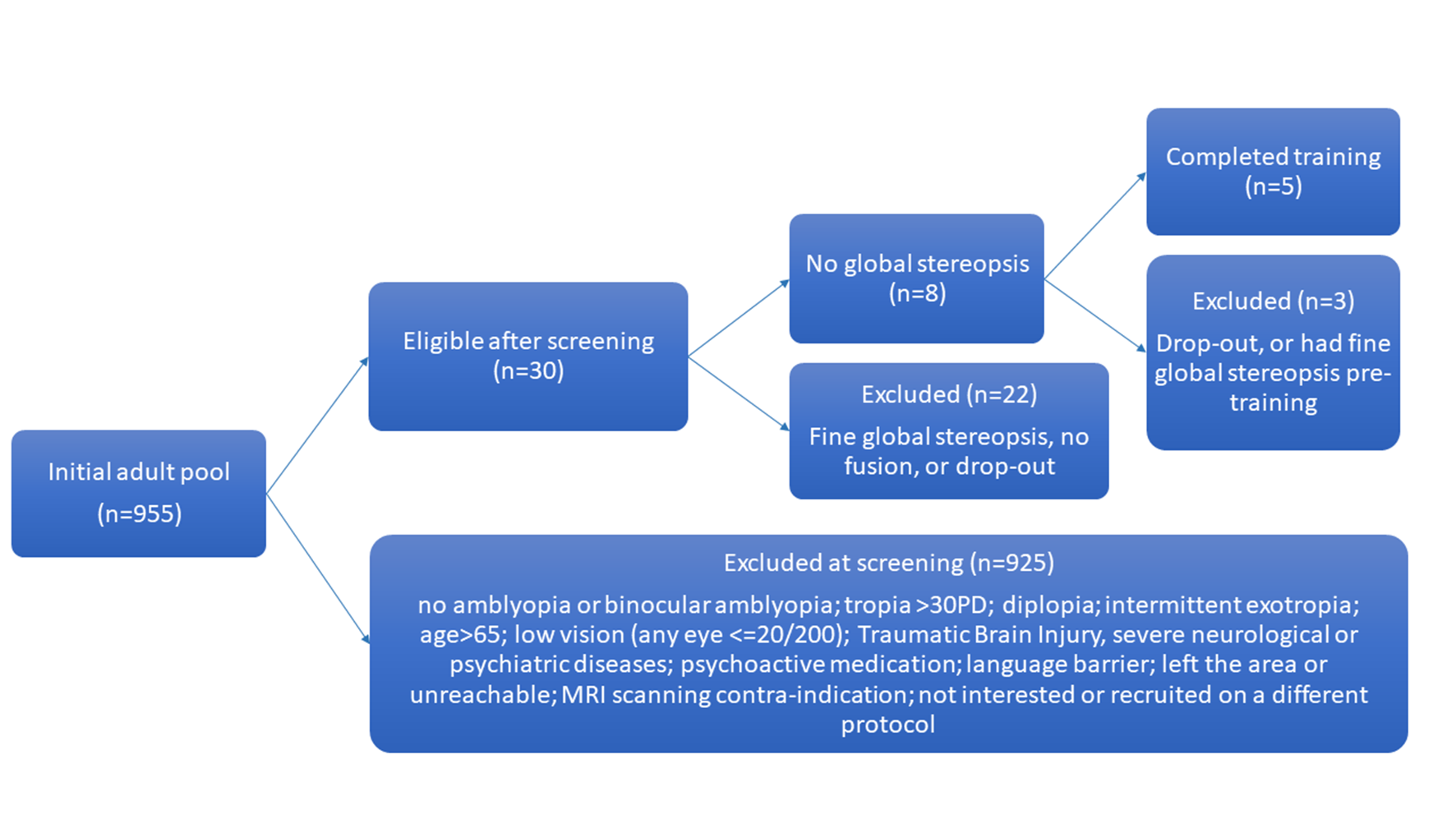

Supplement: Supplementary Figure 1 — Diagram of the recruitment procedure and results. [file Image_1.PNG]
